# The Impact of Top-Down Attention on Emotion Ensemble Perception: Fear-Guided Attention Leads to Cautious Decisions

**DOI:** 10.1007/s42761-025-00323-y

**Published:** 2025-08-23

**Authors:** Hilary H. T. Ngai, Jingwen Jin

**Affiliations:** 1 Department of Psychology, The University of Hong Kong, Hong Kong SAR, China; 2 State Key Laboratory of Brain and Cognitive Sciences, The University of Hong Kong, Hong Kong SAR, China

**Keywords:** Attention, Emotion, Decision-making, Drift diffusion model, Ensemble perception

## Abstract

Accurately deducing the emotional tenor of our visual surroundings has important repercussions, such as whether to approach a friendly group or flee a threatening mob. Perceptual decisions regarding individual emotional stimuli are heavily influenced by both task-driven (top-down) and stimulus-driven (bottom-up) attention. However, the impact of these attentional factors on perceptual decisions regarding the overarching or “gist” emotion conveyed by an ensemble of emotional stimuli remains unclear. We manipulated top-down and bottom-up attention in a task in which participants (*N* = 95) judged the average emotion of ensembles and applied drift diffusion modeling to uncover the underlying computational mechanisms. Our results showed different effects of attention, driven by task-related top-down compared to stimulus-related bottom-up factors, on overall ensemble perception. The inclusion of extremely fearful stimuli (bottom-up effect) led to (1) the ensemble being perceived as fearful more frequently and faster and (2) more efficient fearful evidence accumulation, allowing one to reach a fearful decision faster. In contrast, making fear-related decisions (top-down effect) led to (1) the ensemble being perceived as fearful less frequently and slower with a (2) wider boundary separation, indicating more evidence required for making a fearful decision. These distinct effects are unique to fear. Our findings provide important knowledge in understanding the interplay of top-down and bottom-up attentional mechanisms when swiftly integrating multiple sensory emotional inputs into a coherent perceptual experience, which carries significant implications across social and clinical contexts.

**Supplementary Information:**

The online version contains supplementary material available at 10.1007/s42761-025-00323-y.

We often encounter emotional information in complex visual environments filled with multiple stimuli. Vision science studies show that our visual system can extract summary or “gist” information from a multi-stimuli information space in a process referred to as ensemble perception, enabling us to form a categorical judgment about the overall impression (Alvarez, 2011; Whitney & Yamanashi Leib, 2018). Similarly, humans can accurately extract the mean emotion or the emotional “gist” across a set of faces (Baek & Chong, 2020; Li et al., 2016; Wolfe et al., 2015). While ensemble perception itself is generally considered to be an efficient process that operates even when attention is limited (Alvarez & Oliva, 2008; Bronfman et al., 2018), research is increasingly showing that attention can also strongly modulate it (Chong & Treisman, 2005; de Fockert & Marchant, 2008; Whitney & Yamanashi Leib, 2018).

The way attention influences the integration of information across stimuli of varying emotional intensities, leading to an ultimate decision, still lacks clarity. How are decisions regarding an emotion ensemble shaped by the interplay of stimulus-driven bottom-up and task-driven top-down attention? When making an overall decision regarding the emotion of a crowd of faces, how does the presence of some very threatening faces affect our perception of the overall emotion? How does purposeful vigilance towards threatening faces further affect our decision-making? Moreover, what is the computational nature of these impacts on decision-making? Is trait anxiety associated with such processes? Answers to these questions would inform fundamental aspects of how humans perceive and interact adaptively with their complex emotional environments.

Emotion-related attention can be driven by two factors. It is proposed that, due to their saliency, emotional stimuli capture attention exogenously in an automatic, bottom-up manner (Brosch et al., 2010), while emotion-related expectations, goals, and contexts guide attention in a top-down manner (Mohanty et al., 2023; Sussman, Jin et al., 2016). Traditionally, emotion theories have emphasized that due to their evolutionary value, emotional stimuli are prioritized in information processing (Ledoux, 1998; Pessoa, 2005; Wang et al., 1994). For instance, negative stimuli, particularly fearful stimuli and, to a lesser extent, positive (e.g., happy) stimuli are detected faster. Fearful stimuli “pop out” compared to non-fearful ones and capture attention automatically (Adolphs, 2008; Öhman et al., 1995). Empirically, studies rooted in this exogenous perspective tend to utilize paradigms in which emotional stimuli are distractors and their detection is task-irrelevant in order to highlight that the attention-capturing effect is automatic and involuntary (Brown et al., 2020; Delchau et al., 2020; Vuilleumier, 2015).

Recent theoretical advancements and surging empirical evidence in affective science have shifted focus from bottom-up, exogenous factors to intraindividual factors that can exert top-down influences on emotion-related perception and judgment (Mohanty & Sussman, 2013; Mohanty et al., 2023; Zadra & Clore, 2011). Even the effects that are typically considered automatic and bottom-up are sensitive to top-down influences (Brown et al., 2020; Frischen et al., 2008; Hahn & Gronlund, 2007; Vromen et al., 2016). According to the theory of constructed emotion, the predictive processing framework, and the emotion appraisal theory, emotion perception can be influenced by top-down factors, such as goal relevance, as well as what the viewer expects and attends to (Feldman Barrett, 2011; Fournier & Koenig, 2024; Friston, 2010; Rao & Ballard, 1999; Scherer & Moors, 2019). Research rooted in this top-down perspective stresses that detection and perception of emotional stimuli often occur in contexts where prior knowledge and contextual cues help generate the hypotheses about what sensory signals are relevant and likely, guiding sensory evidence matching and influencing perception (Baluch & Itti, 2011; Dosher & Lu, 1999; Mohanty et al., 2023). In terms of paradigm, these studies have employed tasks that demand explicit emotion identification or discrimination (Imbriano et al., 2020; Mohanty et al., 2009; Sussman et al., 2017; Sussman, Jin et al., 2016; Sussman, Szekely et al., 2016). Hence, attention in these tasks is endogenously guided towards an emotional target. By comparing performance between different emotion-guided attention conditions (e.g., fear-guided versus neutral-guided), researchers examine how such top-down attention modulates the perception of emotional stimuli. Top-down attention, manipulated by task relevance, has been shown to sharpen sensitivity to the attended feature or object, enhancing the efficiency of processing target-congruent signals (Corbetta & Shulman, 2002; Mohanty et al., 2023; Nook et al., 2015; Treisman, 2013). It has also been shown that top-down attention sharpens the distinction between anticipated and unanticipated percepts in the visual brain (Jiang et al., 2013; Summerfield & Egner, 2009). In affective science research, empirical evidence demonstrates that attending to fearful (compared to neutral) stimuli enhances both the speed and the perceptual sensitivity in discriminating fearful from neutral stimuli (Ozturk et al., 2023; Sussman et al., 2017; Sussman, Jin et al., 2016; Sussman, Szekely et al., 2016).

While existing research on bottom-up and top-down factors so far sheds light on the interplay of these factors in guiding perception of single stimuli, the mechanisms by which these factors guide perception of an ensemble containing emotional stimuli remain unclear. In addition, reaction time (RT) and choice can summarize the decision-maker’s performance; however, these measures alone are limited in providing insight into the computational mechanisms. Drift diffusion model (DDM) which takes the composite of both RT and choice data, provides a way to computationally elucidate how bottom-up and top-down emotional information is integrated in the service of decision-making (Wiecki et al., 2015). DDM has been used to examine the components of cognitive processing in two-alternative decision-making tasks with multiple stimuli (Ratcliff, 1978; Ratcliff & McKoon, 2008) and single emotional stimuli (Ozturk et al., 2023). According to DDM, a perceptual decision is formed when accumulated noise-collapsed sensory evidence is sufficient to pass a decision threshold. Here, evidence accumulation starts at a baseline (or starting point); the efficiency of evidence accumulation is coded in drift rate; and the amount of evidence needed to differentiate between two decision options is reflected in the boundary separation. Bottom-up stimulus properties, including emotional aspects, can affect the drift rate, whereas top-down factors may influence the starting point, boundary separation, and drift rate (Dunovan et al., 2014; Kloosterman et al., 2019; Ozturk et al., 2023; Urai et al., 2019).

In the present study, we applied DDM to behavioral performance on an emotion ensemble task in which participants viewed faces depicting varying expressions ranging from fearful to happy. The bottom-up stimulus evidence was manipulated by omitting or including extreme emotional expressions within the ensemble. Following an established approach, top-down emotion-guided attention was manipulated using task relevance (Jiang et al., 2013; Summerfield et al., 2006; Sussman, Jin et al., 2016; Sussman, Szekely et al., 2016). Specifically, participants’ attention was directed towards fearful features when judging whether the ensemble stimuli were “fearful or not” and directed towards happy features when judging whether the ensemble stimuli were “happy or not.”

Regarding the stimulus-driven bottom-up effect, we hypothesized that the presence of extreme emotional stimuli within an ensemble would capture attention and shift the categorical decision towards the corresponding emotion category. Computationally, this stimulus-driven effect would be reflected in higher drift rate towards the corresponding emotion decision boundary because the presence of extreme emotional stimuli increased the corresponding emotional evidence. Then, based on findings in single-stimulus experiments, top-down attention towards emotional stimuli might further augment the bottom-up effect, resulting in greater overall judgments of the ensemble as the attended emotion. Computationally, this top-down influence would reflect greater drift rate towards the emotion decision boundary, or greater shift in the starting point of evidence accumulation towards the emotion boundary, or a lower decision threshold for identifying the ensemble as the target emotion. Lastly, as a secondary research question, we also explored whether trait anxiety is associated with behavioral performance and DDM measures as seen in previous single-stimulus studies (Hartley & Phelps, 2012; Mogg & Bradley, 1998; Ozturk et al., 2023). We hypothesized that individuals with higher anxiety would exhibit exaggerated bottom-up and top-down effects of fear.

## Method

### Participants

This study used participants who were recruited for a bigger project whose aims also included investigating various individual differences on psychopathology. Thus, the sample size of this study was determined based on power analyses considering both within- and between-subjects effects. A within-subject repeated measures analysis of variance (rmANOVA) test with a power of .8, effect size of 0.3, and alpha = 0.05 yielded a minimal sample size of 34; a *t*-test with the same power, effect size, and error rate on individual differences in psychopathology yielded a total sample size of 90; hence, the larger was chosen as preregistered (Faul et al., 2007). To clarify, the present study primarily focuses on the within-subject effects of emotion and secondarily on anxiety-related psychopathology. Using a sample size much larger than the minimal sample size increases the confidence of the results reported below.

One hundred participants (*mean age* = 24.1 years old, *SD* = 6.6; female = 76%, male = 24%; Chinese and other Asian = 99%) from the University of Hong Kong (HKU) were recruited at the end of 2022 for the present study. Data from five participants were discarded due to poor data quality (i.e., falling asleep). Thus, the final dataset consisted of 95 participants. A sensitivity analysis was conducted to estimate the smallest effect size detectable with the sample size and analysis design (Faul et al., 2007). Assuming an alpha level of .05 and power of .80, the analysis indicated that the study could reliably detect effect sizes as small as Cohen’s *f* = 0.0942. This suggests our study design and sample size provide sufficient sensitivity to identify small but meaningful effects. All participants provided written informed consent, reported fluency in English (the educational language in HKU), and had normal or corrected-to-normal vision. The study was approved by the Human Research Ethics Committee of HKU. Participants were compensated 75 HKD for their participation.

### Procedure and Experimental Design

**Fig. 1:**
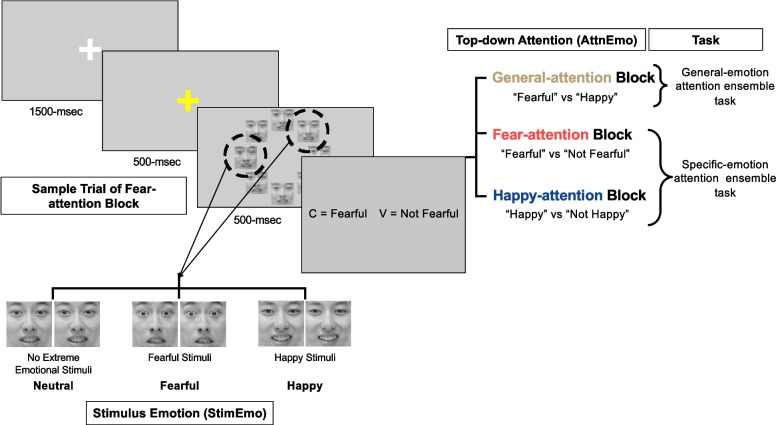
Task paradigm with sample trial and conditions. *Note.* An example of a trial with 8 faces (i.e., the emotion ensemble) presented in an annulus where the condition of StimEmo was manipulated by having no extreme emotional stimuli (neutral), or replacing two random faces with fearful stimuli (fearful) or happy stimuli (happy). In the general-emotion attention ensemble task, participants discerned the mean emotion of the 8 faces under general-attention by deciding between “Fearful” and “Happy.” Top-down attention was also manipulated in the specific-emotion attention ensemble task using fear-attention and happy-attention guided instructions

Participants were asked to complete two computer tasks—the general-emotion attention ensemble task and the specific-emotion attention ensemble task (Fig. 1), which the participants all completed in the same order. Both computer tasks were delivered using PsychoPy (Version 3.2.4). As part of the bigger project mentioned above, all participants also completed a battery of questionnaires after the two computer tasks.

#### Stimuli

Ten face images (five fearful, five happy) from five actors (2 female, 3 male) in the cited studies were used (Hsiao et al., 2021; Zhang et al., 2019). This database was chosen to match the demographics of the participants (Barrett et al., 2011). Fearful and happy faces were selected as they were emotions of high arousal, while having opposite emotional valences (Russell, 1980; Watson et al., 1999). In line with established methods in emotion ensemble research, which frequently employ morphing between emotions of differing valences, such as fear and happiness (Elias et al., 2017; Haberman & Whitney, 2007; Ji et al., 2018), these emotions were selected to investigate how opposing motivational states impact decision-making. It is important to note, however, that fear and happy expressions were not treated as direct opposites (i.e., fundamentally contrary in nature) but instead as representing unique ends of an emotion spectrum (Adolph & Alpers, 2010). While anger and fear both fall under the category of negative valence, angry faces were excluded from the study due to their potential to induce behavioral changes in the target of the anger (e.g., the participants). This exclusion was made to avoid eliciting a more intricate response, as anger can evoke complex reactions (Pichon et al., 2009). Using greyscale fearful and happy images of each actor as anchors, a spectrum of 99 images ranging from extremely fearful to extremely happy was created using Fantamorph (Abrosoft Fantamorph Version 5.6.2; www.fantamorph.com). The resulting 101 images for each actor were numerically encoded from 0 to 100 reflecting their emotional value (EV). For example, EV of 0 referred to the anchoring fearful image while EV of 100 referred to the anchoring happy image. Lastly, the images were equalized for luminance and spatial frequency using the SHINE (Spectrum, Histogram and Intensity Normalization and Equalization) toolbox for MATLAB (Willenbockel et al., 2010).

#### The General-Emotion Attention Ensemble Task

To establish a baseline condition in which participants’ attention was not encouraged to focus on either fearful or happy target endogenously, participants first completed a decision-making task, wherein they decided whether the presented emotion ensemble was overall more fearful or happier (*general* AttnEmo condition). As displayed in Fig. 1, each trial began with a white fixation cross for 1500 ms, followed by a yellow cross for 500 ms, signaling the upcoming arrival of the faces. Then, the set of 8 faces—the emotion ensemble—were presented in an annulus for 500 ms. Upon the offset of the emotion ensemble, participants decided whether the emotion ensemble, on average, was fearful or happy by pressing one of two buttons as quickly and accurately as possible.

To examine the effects of extreme emotional stimuli on the average judgment of emotion ensembles, three stimuli conditions were used. First, the eight faces per trial were generated by drawing eight images of distinct EVs from a truncated Gaussian distribution. In the condition with no extreme emotional stimuli, the faces had a mean EV of 50 (i.e., the midpoint of the fearful-to-happy EV spectrum), a standard deviation of 10 EV, and were bounded between 30 and 70 EV to create a homogenous looking array of faces with mild expressions. This condition was named the *neutral* stimulus emotion (StimEmo) condition, denoting that neither fearful nor happy emotion was dominant, implying a chance of choosing either option equally. Second, in the StimEmo condition with extremely *fearful* stimuli, two randomly selected faces from the neutral condition were replaced by very fearful faces (EV = 0 and 10). Finally, in the StimEmo condition with extremely *happy* stimuli, two randomly selected faces were replaced by very happy faces (EV = 90 and 100). Overall, the mean EV across the eight faces of a trial was close to 50 for the neutral StimEmo condition, in the lower EV range for the fearful condition and higher EV range for the happy condition (see Supplementary Table 1 and Supplementary Fig. 1). Since emotion perception is largely subjective (Barrett et al., 2011; Brosch et al., 2010), the neutral StimEmo condition was created to explore how a decision would be made in a context with no clear or accurate answer.

The locations of the eight faces on the annulus were randomly assigned in each trial. There were 100 trials in each of the conditions. The trials were pseudorandomized such that the same face actor and StimEmo condition were not presented more than three trials in a row, to avoid local learning effects. Presenting faces from the same actor minimizes the influence of potential confounding factors such as attractiveness and gender on attention and emotion expression evaluation (Whitney & Yamanashi Leib, 2018).

#### The Specific-Emotion Attention Ensemble Task

To investigate how top-down attention directed towards a particular emotion influences average judgments of emotion ensembles, we adopted an “X or Not X” task design well established in previous single-stimulus research examining top-down, endogenous attention (Imbriano et al., 2020; Sussman, Jin et al., 2016; Sussman, Szekely et al., 2016). In this task **(**Fig. 1**)**, participants were presented with the same emotion ensemble stimuli as described in the general-emotion attention ensemble task but were instructed to make a “Fearful or Not Fearful” decision in the *fear*-attention blocks and a “Happy or Not Happy” decision in the *happy*-attention blocks. The total number of trials doubled compared to the general-emotion attention ensemble task. The fear- and happy-attention blocks alternated, and the sequence was counterbalanced across participants.

#### Battery of Questionnaires

Individual differences in anxiety were measured by administering questionnaires on Qualtrics. Included questionnaires were Penn State Worry Questionnaire (PSWQ, (Meyer et al., 1990)) which measures anxious apprehension manifesting in worry, and the Depression Anxiety Stress Scale (DASS-21, (Lovibond & Lovibond, 1995)) which includes a subscale measuring the presence and intensity of symptoms related to trait anxiety. Inventory of Depression and Anxiety Symptoms (IDAS-II, (Watson et al., 2007)) was also administered but not analyzed along with the above questionnaire’s components involving depression, as such components are aimed to be addressed as part of the larger project.

### Data Analyses

#### Behavioral Analyses

Combining the two tasks allowed us to examine overall perceptual decision regarding emotion ensembles affected by bottom-up Stimulus Emotion (StimEmo: neutral, fearful, happy) and top-down Attention to Emotion (AttnEmo: general, fear, happy).

Proportion of “Fearful” decisions in the general- and fear-attention conditions was computed, as well as “Not Happy” decisions in the happy-attention condition. It is worth noting that only these proportions were computed, as they were complementary to their opposing choices of “Not Fearful” and “Happy.” Then, to examine the effects of StimEmo and AttnEmo on decision-making, two 3 (StimEmo: neutral, fearful, happy) × 3 (AttnEmo: general, fear, happy) rmANOVAs were conducted for choice and RT separately as preregistered. Note that the proportion of decisions should not be considered a measure of decision accuracy as emotions are subjectively perceived based on many contextual factors (Barrett, 2006; Barrett et al., 2011). What matters are the potential differences in the proportion of decisions between different task conditions.

#### Hierarchical Drift Diffusion Modeling

To gain insight into the computational components affected by bottom-up StimEmo and top-down AttnEmo, the hierarchical drift diffusion model (HDDM) was applied as a non-preregistered exploratory analysis (Wiecki et al., 2013). The full technical details of the HDDM procedure are included in the Supplementary materials; here, we describe the key components. For the analysis, trials with RTs greater than 4000 ms were excluded (number of trials excluded = 443, 0.52%). This resulted in a total of 85,057 valid trials across all participants. The modeling was conducted in the Jupyter Notebook environment (v 6.1.4). It is important to note that this study was part of a larger project with the primary aim of investigating various individual differences in anxiety and depression-related psychopathology. As such, the preregistration for this study was written with the broader scope of the project in mind. The present manuscript, however, primarily focuses on the within-subject effects of emotion and secondarily on anxiety-related psychopathology measures; hence, this manuscript includes exploratory DDM analyses that were not preregistered.

**Fig. 2:**
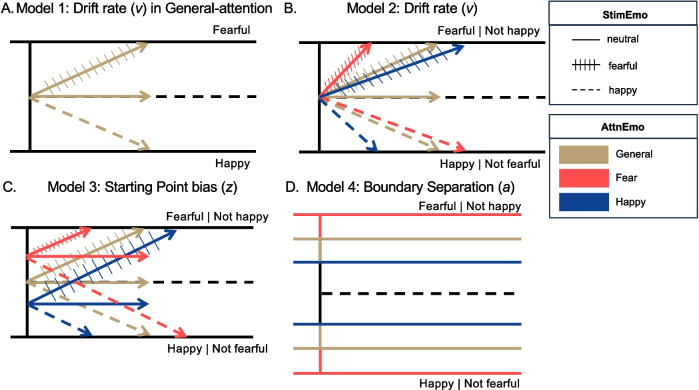
HDDM models schematics. *Note*. **A** Model 1 DDM schematic, which allowed the drift rate to vary by StimEmo in general-attention trials. The upper boundary was “Fearful” while the lower boundary was “Happy,” with a starting point located equidistant between the two boundaries. Next, AttnEmo affected **B** drift rate in Model 2, **C** starting point in Model 3, and **D** boundary separation in Model 4. Models 2–4 were applied to the general- and fear-attention trials, then the general- and happy-attention trials separately

Model specifications were constructed based on our research questions and hypotheses with illustrations in Fig. 2. If the participants were sensitive to evidence from the extreme emotional stimuli, then the ensembles with fearful and happy StimEmo would have faster drift rates (denoted as *v*) towards the corresponding decision boundary compared to the neutral condition. To test this hypothesis, we constructed Model 1 and tested it on trials from the general-emotion attention ensemble task (Fig. 2A). The upper decision boundary represented the fearful decision, and the lower decision boundary represented the happy decision regarding the ensemble. Accordingly, a positive drift rate would indicate evidence accumulation towards the fearful boundary, and a negative drift rate would indicate evidence accumulation towards the happy boundary. For this task, the instructions were consistent across all conditions, and the StimEmo condition was not known to the participant. Hence, a starting point located equidistant between the upper and lower decision boundaries was specified, and boundary separation and the non-decision time (denoted as *T*_*er*_) were estimated at the subject level. Only drift rate was allowed to vary by the StimEmo condition.

The second question was how top-down attention affects the decision regarding the ensemble. We constructed three models testing three competing computational mechanisms based on previous literature and current behavioral results. Previous research has shown that top-down attention can bias the decision-making process by altering drift rate towards the decision boundary, shifting the starting point closer towards the attended decision boundary, and decreasing the boundary separation requiring less evidence for the decision (Dunovan et al., 2014; Krajbich et al., 2010; Mohanty et al., 2023; Mulder et al., 2012; Ozturk et al., 2023). One parameter was chosen to vary in each model to maintain parsimony in order to isolate the specific impact of each parameter on the decision-making processes and to avoid overfitting. Thus, we tested whether the impact of AttnEmo was better explained by a change in drift rate (*v*), a shift of the starting point bias (*z*), or a change in boundary separation (*a*).

Model 2 allowed the drift rate to vary as a function of AttnEmo (Fig. 2B), Model 3 allowed the starting point to vary as a function of AttnEmo (Fig. 2C), and Model 4 allowed the boundary separation to vary as a function of AttnEmo (Fig. 2D). Models 2, 3, and 4 allowed the drift rate to vary as a function of StimEmo. We compared Models 2–4 one time using trials from the general- and fear-attention conditions, with the upper boundary being the fearful decision and the lower boundary as happy/not fearful decision. We compared Models 2–4 another time using trials from the general- and happy-attention conditions, with the upper boundary being the happy decision and the lower boundary as fearful/not happy decision. Separate models were fitted for the fear-attention and happy-attention conditions to examine and distinguish their individual effects.

Each model was evaluated by drawing 5,000 iterations, with a burn-in of 2,500 samples for chain stabilization (Gelman et al., 2004), and a thin of five. Model fit was evaluated using the deviance information criteria (DIC), which considers both goodness-of-fit and model complexity (Spiegelhalter et al., 2002). The model with the lower DIC value among all tested models was selected as the best-fitting model. For the best-fitting model, model convergence was assessed visually and quantitatively through the Gelman-Rubin R-hat statistic (Brooks & Gelman, 1998). Lastly, hypothesis testing was performed by analyzing the parameter estimates of the winning model with a critical value (alpha) of 0.05. Accordingly, the results were declared as statistically significant if *q* was < .025 for a two-tailed test (Cavanagh et al., 2011). The *q*-value quantifies the probability that the DDM parameter is larger in one condition relative to another, derived from the proportion of posterior distributions favoring this directional difference.

Posterior predictive checks (PPC) and parameter recovery analyses were conducted for the winning models (refer to Supplementary Methods & Results for detailed procedures and results).

#### Association with Anxiety

Lastly, as a preregistered secondary research question, we also examined the potential effects of trait anxiety in the context of the current tasks. Anxiety measures from the questionnaire data were extracted to investigate any relationship with the behavioral and HDDM measures. Specifically, scores of (1) PSWQ (anxious apprehension) and (2) DASS-21 (trait anxiety) were entered as covariates, separately, in the above rmANOVAs.

### Statements and Declarations

We preregistered the experiment on the Open Science Framework (OSF) prior to data analysis but after data collection. Preregistrations, materials are made available on the OSF at https://osf.io/3yvb4. Data can be made available upon request to the corresponding author. Given the scope of the current manuscript, some preregistered hypotheses were not tested in the current manuscript but will be addressed in future manuscripts. During manuscript editing, ChatGPT 3.5 available through The University of Hong Kong (https://chatgpt.hku.hk) was occasionally used to check grammar (OpenAI, 2023). We report how we determined our sample size, all data exclusions (if any), all manipulations, and all measures in the study.

## Results

### Bottom-Up and Top-Down Factors Affect Choice Differently

**Fig. 3:**
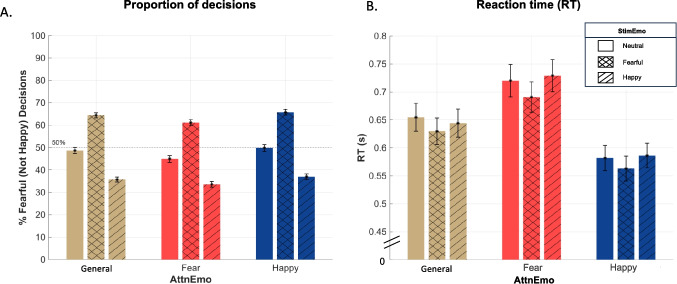
Behavioral results of choice and RT. *Note.* **A** Mean proportions of “fearful” decisions. This also includes “not happy” decisions in the happy AttnEmo blocks. Overall, bottom-up effect of StimEmo conditions was observed, indicating that the presence of extreme stimuli resulted in a higher likelihood of making decisions in favor of those stimuli. In contrast, the top-down effect of AttnEmo conditions revealed a conservative threshold in making a fear-present decision under fear-attention. **B** Mean RTs in the StimEmo and AttnEmo conditions. Presence of extremely fearful stimuli led to faster RT. On the other hand, top-down fear-attention led to slower RT. Error bars denote standard error. Individual datapoints for decision and RT data across all participants and all conditions are shown in Supplementary Fig. 4

Effects of bottom-up StimEmo and top-down AttnEmo on the judgment of emotion ensembles (Fig. 3A) demonstrated that participants were sensitive to extreme emotional evidence in the ensemble (*F*(2,188) = 697.37, *p* < .001, η_p_^2^ = 0.881, 90% CI [0.856; 0.897]). Fearful StimEmo led to more fearful decisions (*M* = 63.8%, *SD* = 1.1%) than neutral StimEmo (*M* = 47.8%, *SD* = 1.2%), and the least proportion of fearful decisions was made in the happy StimEmo condition (*M* = 35.4%, *SD* = 1.0%). Follow-up analyses were conducted using Tukey’s post hoc test, revealing significant differences between conditions: fearful vs. neutral (*t*(94) = 23.90,* p* < .001, *d* = 1.96) and neutral vs. happy (*t*(94) =  − 20.40, *p* < .001, *d* = 1.72).

Top-down AttnEmo also impacted the ensemble decision (*F*(2, 188) = 7.45, *p* < .001, η_p_^2^ = 0.073, 90% CI (0.020; 0.133)), with the results contrary to our hypothesized direction. Compared to the general-attention condition (*M* = 49.6%, *SD* = 1.1%), participants judged the ensemble as being fearful less frequently under fear-attention (*M* = 46.5%, *SD* = 1.3%, *t*(94) = 2.74, *p* = .02, *d* =  − 0.28). The proportion of fearful choices under fear-attention was also lower than the “not happy” choices under the happy-attention condition (*M* = 50.8%, *SD* = 1.2%, *t*(94) =  − 3.52, *p* = .002, *d* =  − 0.36). Together, the results indicated a conservative threshold in making a fear-present decision under fear-attention. There were no differences in overall decisions between the general-attention and happy-attention conditions (*p* = .50). No interaction between bottom-up StimEmo and top-down AttnEmo was revealed (*p* = .36).

### Bottom-Up and Top-Down Factors Affect Reaction Time Differently

Similar to the choice results, rmANOVA (Fig. 3B) showed main effects of StimEmo (*F*(2,188) = 10.82, *p* < .001, η_p_^2^ = 0.103, 90% CI [0.040; 0.170]) and AttnEmo (*F*(2, 188) = 25.40, *p* < .001, η_p_^2^ = 0.213, 90% CI [0.128; 0.289]) on RT, with no significant interaction (*p* = .47). The presence of extremely fearful stimuli in the ensemble (*M* = 628 ms, *SD* = 21.3 ms) led to a faster overall ensemble decision than the presence of extremely happy (*M* = 653 ms, *SD* = 22.4 ms, *t*(94) =  − 4.18, *p* < .001, *d* =  − 0.43) and neutral stimuli (*M* = 652 ms, *SD* = 22.5 ms, *t*(94) =  − 3.87*, p* < .001, *d* =  − 0.40), with the latter two not differing significantly (*p* = .99). In contrast, under fear-attention, RT was slower (*M* = 713 s, SD = 27.5 ms), compared to general-attention (*M* = 643 ms, *SD* = 23.8 ms, *t*(94) = 3.04, *p* = .003, *d* = 0.31), which in turn was slower than under happy-attention (*M* = 577 ms, *SD* = 21.7 ms*, t*(94) = 3.62, *p* < .001, *d* = 0.37). Follow-up analyses showed that the slower RT under fear-attention compared to happy-attention applied to both “target present” and “target absent” trials (see Supplementary results).

Overall, behavioral results indicated that the presence of extremely fearful elements in an ensemble led to it being judged as fearful more frequently and faster, suggesting a bottom-up effect. In contrast, top-down fear-attention led to the ensemble being judged as fearful less frequently and more slowly. This was different than happy-attention, which hastened overall emotion decisions regarding the ensemble.

### Extreme Emotional Stimuli Enhance Drift Rate

**Fig. 4:**
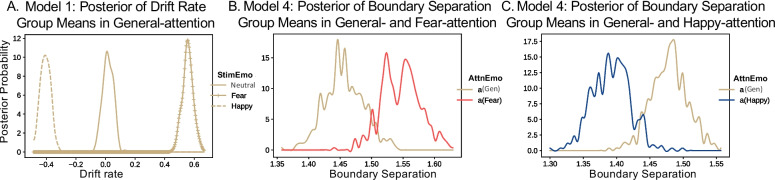
DDM results. *Note.* **A** The posterior group mean of the drift rate of different StimEmo conditions during the general-emotion attention ensemble task. This shows that drift rate was most positive for “fearful” StimEmo and most negative for “happy” StimEmo. **B** The best-fitting model of having boundary separation vary by AttnEmo (Model 4) revealed that *a* was larger for fear-attention trials compared to the general-attention condition and that **C** *a* was smaller for happy-attention trials. The best-fitting models’ convergence was assessed visually. Models 1 and 4 all showed that Gelman-Rubin $\widehat{R}$ values were close to 1 and not larger than 1.1, indicating that the Markov chain Monte Carlo (MCMC) chains converged successfully

To examine the computational mechanisms underlying behavioral results, HDDM (Wiecki et al., 2013) was used to estimate the effects of extreme emotional stimuli on the drift rate (*v*). As seen in Fig. 4A, in the general-emotion attention ensemble task, the drift rate was most positive (i.e., towards fearful decision boundary) when extremely fearful stimuli were included in the ensemble and most negative (i.e., towards happy decision boundary) when extremely happy stimuli were included in the ensemble (group-level mean *v*_fear_ = 0.55, *v*_neutral_ = 0.02, *v*_happy_ =  − 0.40; *q* < .001 for all pair-wise comparisons). Hence, the presence of extremely fearful or happy evidence in the ensemble led to a higher rate of evidence accumulation for the corresponding decision. Additionally, the drift rate was faster in the case of fearful as compared to happy StimEmo in the ensembles (*q* < .001), indicating that the evidence accumulation was more efficient in the presence of extremely fearful than extremely happy stimuli.

### Top-Down Fear-Attention Enlarges Boundary Separation

To examine the effects of top-down attention on the decision-making process, we examined three models (M2-4). Model comparison showed that the model in which boundary separation varied by AttnEmo (DIC_M4_ = 111,564 < DIC_M3_ = 113,813 < DIC_M2_ = 117,340) approximated the data from the general- and fear-attention conditions best compared to starting point and drift rate with adequate model convergence. Examining the estimated boundary separation parameters revealed that the boundary separation was larger for fear-attention trials, indicating more evidence was needed to make a “fearful” vs “not fearful” decision compared to when making a “fearful” vs “happy” decision (*a*_General_ = 1.46, *a*_F_ = 1.55, *q* < .02) (Fig. 4B). The same analysis was conducted on trials from general- and happy-attention conditions. Model comparison showed that here too, in the best-fitting model, boundary separation varied by AttnEmo (DIC_M4_ = 101,702 < DIC_M3_ = 103,595 < DIC_M2_ = 107,279), with adequate model convergence. In contrast to the fear-attention, boundary separation was smaller for happy-attention, indicating less evidence is needed when making a “happy” vs “not happy” decision as compared to when making a “happy” vs “fear” decision (*a*_General_ = 1.48, *a*_H_ = 1.40, *q* < .004) (Fig. 4C). Posterior predictive checks (PPC) and parameter recovery results of winning models are outlined in Supplementary Results.

### Trait Anxiety Does Not Affect Emotion Ensemble Perception

To identify whether anxiety modulated the behavioral measures of decision and RT as well as HDDM measures, ANCOVA was applied. However, there was no significant effect of any anxiety measures (*p*_all_ > .05) (see Supplementary results).

## Discussion

How do humans judge the emotional tone of a complex scene? In the present study, we investigated how emotion-related bottom-up and top-down influences shape overall judgments of emotion ensembles. The presence of extremely fearful stimuli in the ensemble led to faster and more frequent mean judgments of fear for the overall ensemble. In comparing fear-related and non-fear-related decisions, contrasting findings emerged. Firstly, top-down attention towards fear led to a decreased frequency and slower perception of the ensemble as fearful. Secondly, through DDM, there was evidence of wider boundary separation, suggesting caution or a greater need for information to make fearful decisions. Notably, these contrasting effects are specific to fear, highlighting the distinct impact fear-related decisions have, as compared to happy-related decisions.

The bottom-up stimulus effect of extreme emotional evidence indicated that humans incorporate the extreme evidence when computing summary judgments (Bannerman et al., 2009; Desimone & Duncan, 1995). This is in direct contrast with some existing findings in ensemble perception of emotional stimuli, where extreme pieces were considered outliers and were downweighted in participants’ decisions (Haberman & Whitney, 2010). However, it is to be noted that the outliers in those studies refer to faces that deviated from the ensemble’s mean largely (e.g., ± 3SD from mean) while the extreme emotional faces in the current task are not considered outliers. Recent findings have also pointed to other factors, such as spatial distribution of stimuli, impacting the rejection of outliers during ensemble perception (Lee & Chong, 2024). Moreover, the participants were clearly instructed to average across all faces. Additionally, the HDDM went beyond behavioral data by offering additional insights into the underlying computational mechanisms of this decision-making process (Wiecki et al., 2015). The results suggested that this behavioral effect can be explained by faster accumulation of evidence in favor of the fearful decision, implying a mechanism by which the saliency of the fearful stimuli influences emotion ensemble perception. This is in line with basic non-affective science findings such as the random dot motion perception, in which a higher strength stimulus (with more dots moving in the same direction) can increase the drift rate, making it easier to perceive the direction of motion (Britten et al., 1993; Gold & Shadlen, 2007; Lee & Usher, 2023). This finding is also consistent with the classical hypothesis that exogenously threatening signals are captured faster as they are prioritized in bottom-up processing, purportedly due to a fast magnocellular pathway reaching the amygdala (Méndez-Bértolo et al., 2016; Pessoa & Adolphs, 2010; Vuilleumier et al., 2003). However, it is important to note that attentional capture is not exclusive to threat-related stimuli (Brosch et al., 2008; Pool et al., 2016). The stronger bottom-up effect for the fear emotion in our findings, as compared to happy emotion, can be attributed to the physical characteristics and the evolutionary significance of threat-related stimuli as well as heightened relevance in certain contexts (Bar-Haim et al., 2007; Barrett, 2006; Larson et al., 2007; Scherer, 2013).

In contrast to the bottom-up effect of fearful stimuli, top-down attention towards fear led to the ensemble being judged as fearful less frequently and slower. HDDM revealed that top-down fear-attention increased the distance between the two decision boundaries. Our results could suggest that fear, as a specific negative emotion, uniquely promotes caution during decision-making by impacting the perceived base rate and threshold of the categories available (Bohil & Maddox, 2001; Johansen et al., 2007; Kahneman & Tversky, 1973). For example, the top-down effects of fear could lead to the labeling of an ensemble with an average expression close to the midpoint as “not fearful” without necessarily categorizing them as “happy.” The slower averaging process under fear-attention, despite the presence of high signal strength in the form of extreme fearful stimuli, can be explained by the interplay between bottom-up and top-down processes. High signal strength typically facilitates faster and more precise averaging (Cha & Chong, 2018; Lee & Chong, 2021; Robitaille & Harris, 2011), and this is observed in the faster drift rate attributable to higher signal in the Fear StimEmo condition. However, the top-down fear-attention condition imposed a higher decision threshold, requiring more evidence accumulation before a decision could be made. In contrast, the null-attention condition, which lacked this top-down modulation, allowed for faster decisions with a lower evidence threshold. Our results may also reflect a speed-accuracy trade-off (Roberts & Hutcherson, 2019; Zhang & Rowe, 2014). Tasks prioritizing accuracy over speed are associated with large boundary separation (Mulder et al., 2013; Ratcliff & Rouder, 1998), whereas time-constrained participants prioritize speed over accuracy, resulting in “collapsed” or reduced boundary separation (Fudenberg et al., 2018). The present findings suggest that participants are more cautious, requiring more evidence and prioritizing accuracy over speed when making decisions under fear-attention (Richards et al., 2014). Interestingly, a striking contrast was observed in the case of top-down happy-attention. The reduced boundary separation in this case suggests that people make hastened decisions, prioritizing speed over accuracy when making decisions under happy-attention. Our findings suggest that attending to fear creates a condition of heightened vigilance, resulting in more careful and deliberate decision-making. This is consistent with suggestions that fear-related attention may produce greater caution for making decisions (Dutilh et al., 2012; Inzlicht et al., 2015). It is important to note that since the physical stimuli are exactly the same across the trials of the three top-down attention conditions, the findings can only be attributed largely to the task instruction, reflecting top-down attention manipulation.

At first glance, it may appear that our ensemble perception findings diverge significantly from findings of single-stimulus tasks. In cases of single stimuli, fear-related attention has been observed to expedite decision-making, amplifying drift rate for fearful stimuli, and biasing the starting point of decision-making towards a fearful decision (Ozturk et al., 2023; Sussman, Jin et al., 2016; Sussman, Szekely et al., 2016). However, it is important to note that in single stimulus studies, fear-related top-down attention also leads to faster drift rate for neutral stimuli, adaptively allowing for better discrimination of fearful and neutral stimuli. Our finding highlights a similar adaptiveness of the emotion-related top-down attention effects on ensemble perception, albeit via different computational mechanisms. In ensemble perception tasks, the key is to integrate evidence from multiple sources. Under such a context, a system that sharpens the signal emanating from just the most emotional source may not be the most adaptive strategy. In such a scenario, top-down fear-attention may lead to a more conservative decision-making approach—requiring more evidence before committing to a choice, which could be deemed a more adaptive approach. Moreover, previous findings show that exogenous attention, driven by salient stimuli, enhances the influence of emotional outliers on ensemble judgments (Son et al., 2023), while endogenous attention, guided by task goals, modulates decision thresholds and precision (Choi & Chong, 2020). Together, bottom-up salience and top-down goals interact to shape how emotional information is integrated and perceived in ensemble perception tasks.

The present findings have implications for our understanding of emotion processing in daily life and psychopathology. An adaptive interaction with our environment requires us to accurately judge the emotional nature of a given scene. In daily life, diverse contexts and societal roles require rapid evaluation of crowd emotions (Alt & Phillips, 2022; Barsade & Gibson, 2012). Examples range from detecting threats in surveillance footage or airport security scans, to assessing the dynamics in a classroom or work meeting. Moreover, due to characteristic hypervigilance towards threat, anxious individuals tend to have heightened attention to fear-related stimuli (MacLeod & Mathews, 1988; Mogg & Bradley, 1998). According to previous research, following acute pharmacological manipulation and stress, rodents exhibit negative bias accompanied by increased cautiousness in decision-making (Hales et al., 2016). This suggests that increased sensitivity to fearful stimuli and boundary separation, or response caution, may be related to anxiety. We speculate that this caution is related to diminished confidence, leading to uncertainty when instructed to make decisions regarding negative outcomes, which is often seen in anxiety (Hartley & Phelps, 2012; Kepecs, 2013). Although the preliminary findings regarding anxiety questionnaire measures from a healthy population yield null results, this paves the way to further explore whether such biases regarding multiple stimuli uncovered in this study also manifest in clinical populations. Doing so will foster a deeper comprehension of the distorted decision-making processes that contribute to anxiety.

There are several limitations to this study that provide directions for future research. Firstly, this study focused on one factor of top-down attention. Other factors such as probability-based expectation and feedback-based learning may be further explored in the future to see how else this process may be altered. For example, previous non-affective science research has shown that expectation about certain events influences perception and decision-making differently than attention (Jiang et al., 2013; Summerfield & Egner, 2009; Summerfield et al., 2006). If signaled that one decision is more probable, expected stimuli are found to be detected quicker, with increased bias (Bar, 2004; De Lange et al., 2018; Stein & Peelen, 2015). Future studies may explore how the ensemble decision-making process may be impacted when the likelihood of stimulus occurrence is explicitly provided. Secondly, the stimuli display time can be varied to examine whether deliberation time would affect the decision-making process. In studies of longer stimuli display time, eye movement may be recorded to assess the effects of selective attention. Moreover, it may be noted that as emotion perception is largely subjective and there was no feedback provided (Feldman, 1995; Lang & Bradley, 2010), “accuracy” may not be objectively meaningful to the participant in this task. In the future, studies can explore learning in a context where feedback is provided, allowing for a more nuanced understanding of how individuals adapt and refine their perception of subjective matters such as emotion. In the current analysis, model construction was clearly guided by existing research and theories. In addition, parsimony was prioritized by allowing only one parameter to vary with task-driven attention in each model to isolate dominant effects and avoid overfitting. Future work could explore models with multiple varying parameters and include recovery analyses to assess their identifiability and robustness. Lastly, the presented stimuli of an ensemble are of the same identity but varying emotions, which is unrealistic. However, the chosen stimuli were carefully controlled for in terms of facial features, attractiveness, and low-level visual factors unrelated to emotions (e.g., contrast, luminance) and to address the dynamic property of facial expressions within one individual. Future studies may aim to extend these findings and answer a different research question by presenting ensembles with multiple identities to increase ecological validity.

In conclusion, this study systematically investigates the top-down and bottom-up influences of attention on emotion ensemble perception. The findings show that bottom-up and top-down emotions, especially fear, exhibit distinct impacts on this decision-making process. The current study enhances our understanding of how the sensory and attention systems integrate salient information in busy environments, shedding light on the cognitive and computational mechanisms involved in emotion-related decision-making.

## Supplementary Information

Below is the link to the electronic supplementary material.

Supplementary file1 (DOCX 1069 KB)

## Additional Information

### Data availability

Sample stimulus, processed data, and code are available for review process upon request. Public link can be generated prior to publication.
