# Supplementary material for: The Impact of Top-Down Attention on Emotion Ensemble Perception: Fear-Guided Attention Leads to Cautious Decisions
Source: Affect Sci. 2025 Aug 23;6(3):534–47. doi: 10.1007/s42761-025-00323-y (PMC12579617; doi:10.1007/s42761-025-00323-y)
Supplement: Supplementary file 1 — Supplementary file1 (DOCX 1069 KB) [file 42761_2025_323_MOESM1_ESM.docx]

**The impact of top-down attention on emotion ensemble perception: fear-guided attention leads to cautious decisions**

Hilary H.T. Ngai^1^, Jingwen Jin^1,2^

^1^Department of Psychology, The University of Hong Kong, Hong Kong SAR, China

^2^State Key Laboratory of Brain and Cognitive Sciences, The University of Hong Kong, Hong Kong SAR, China

**Corresponding: Jingwen Jin, Ph.D.,** [**mailto:**](mailto:)**Frances.Jin@HKU.HK**

**SUPPLEMENTARY INFORMATION**

**Supplementary Method**

**Supplementary Table 1.** *Descriptive statistics of trials in each StimEmo condition.*

| **StimEmo** | **Mean** | **SD** | **Min** | **Max** |
| --- | --- | --- | --- | --- |
| Neutral | 49.97 | 3.40 | 39.25 | 57.75 |
| Fearful | 38.70 | 2.72 | 29.38 | 45.38 |
| Happy | 61.40 | 2.60 | 55.88 | 68.38 |

**Supplementary Figure 1**

*Histogram of the mean EV in each StimEmo condition.*

**Data Analysis**

***Hierarchical Drift Diffusion Modeling (HDDM).*** For DDM analysis only, individual trials with RTs above 4000 msec were excluded (trials excluded = 443, .52%). This resulted in a total of 85,057 valid trials across all participants. The rationale behind choosing 4000-msec as a cut-off point was based on previous work, albeit the cut-off being somewhat arbitrary (Ratcliff, 1985; Ratcliff et al., 1999). Past studies excluded trials above 2500-msec to 5000-msec (De Kock et al., 2021; Lawlor et al., 2020; Son et al., 2019), and 4000-msec was chosen for this current study as it is outside four standard deviations from the mean RT.

HDDM employs hierarchical Bayesian parameter estimation, which deduces the posterior probability density of the DDM parameters generating the observed data for the entire data of participants, while allowing for individual differences (Wiecki et al., 2013). Statistical analysis was performed on the mean group posteriors to evaluate the effect of the set conditions on the DDM parameters (Cavanagh et al., 2011). To answer the second question of whether emotion-guided attention affects starting point or boundary separation, the Deviance Information Criterion (DIC) was used for model comparison. Lower DIC values favor models with highest likelihood and least number of parameters (Gelman et al., 2004).

***HDDM Posterior Predictive Check (PPC) & Parameter Recovery.*** To examine our winning HDDM models, we first conducted posterior predictive checks (PPC), which evaluates how well predictive data generated from sampling posterior parameter values align with the actual data. Secondly, we conducted parameter recovery analyses with known ground-truth parameter values, and examining whether these values can be recovered using the winning models. For Model 1, data was generated using the known ground-truth parameter values while preserving trial counts and condition ratios. The same model structure was then fit to the synthetic data, where drift rate was allowed to vary by StimEmo. For Model 4, the same parameter recovery analysis was conducted twice, once for Fear- and General-attention trials, the second time for Happy- and General-attention trials. The same model structure was then fit to the synthetic data, where drift rate was allowed to vary by StimEmo and boundary separation was allowed to vary by AttnEmo. The recovery analyses were condcuted on dockerHDDM v1.0.1RC (Pan et al., 2025).

***Examining attentional emotion ensemble task choice pattern.*** To gain deeper insight into which types of decisions in the attentional emotion ensemble task drove RT speed, choices were grouped into four types: “Fearful”, “Not Fearful”, “Happy”, and “Not Happy”. Their quantities in each choice type, as well as the respective average RT were calculated. Then, a one-way ANOVA was applied to examine each choice type’s respective RT.

**RT diagnostic analysis.** To evaluate the assumptions of normality and homogeneity of residuals in the repeated measures ANOVA on reaction time (RT), residuals and fitted values were extracted from the ANOVA model object. Diagnostic plots were generated to assess model assumptions: (1) a Q-Q plot was used to visually inspect the normality of residuals; (2) a residuals versus fitted values plot was employed to check for homoscedasticity; and (3) boxplots of residuals by condition were created to evaluate residual distribution across experimental conditions.

**Supplementary Results**

**Follow up analysis of reaction time under top-down attention conditions**

In this follow-up analysis, choice types were investigated to gain deeper insight into which types of decisions in the attentional emotion ensemble task drove reaction time speed. As seen in **Supplementary Figure 2**, one-way ANOVA demonstrated mean RT was significantly different between the four choice types in the task (*F*(3,282) = 47.3, *p* < .001, η_p_^2^=.335). Tukey’s post hoc criterion indicated that the average RT was significantly lower in “Happy” choices (*M* = .572, *SD* = .0224) and “Not Happy” choices (*M* = .588, *SD* = .0229) of the happy-attention condition (*p* < .001), as compared to the “Fearful” choices (*M* = .709, *SD* = .0271) and the “Not Fearful” choices (*M* = .727, *SD* = .0291) of the fear-attention condition (*p* < .001). There was no significant difference between the choices among the fear-attention (*p* = .623) and among the happy-attention (*p* = .625).

In sum, the RT data shows that participants were faster at making happy-related decisions when their endogenous attention was guided toward happy emotion and slower to make fearful-related decisions in the fear-attention block. It took participants similar time to make the two types of decisions among each AttnEmo condition.

**RT diagnostic analysis**

The Q-Q plot of residuals demonstrated that residuals were approximately normally distributed, with points closely aligned along the diagonal reference line and only minor deviations at the distribution tails (**Supplementary Figure 5**). The residuals versus fitted values plot exhibited no discernible pattern, indicating homogeneity of variance across the range of fitted values (**Supplementary Figure 6**). Boxplots of residuals by StimEmo × AttnEmo condition showed residuals centered near zero with similar variability across all conditions (**Supplementary Figure 7**), suggesting consistent model fit without systematic bias or heteroscedasticity.

**Supplementary Figure 2**

*RT data in different types of choices.*

**Supplementary Figure 3**

*Proportion of fearful choices against presented mean EV in all StimEmo conditions.*

**

**

*Note.* The mean emotional values (EV) of trials were calculated by taking the EV of the eight faces in the emotion ensemble and then calculating their average. For each corresponding Mean EV, the proportion of fearful choices were calculated by dividing the number of “Fearful” and “Not Happy” decisions made across all participants over the total number of trials in that condition.

**Supplementary Figure 4**

*Individual datapoints of decisions and RT across all participants and all conditions.*


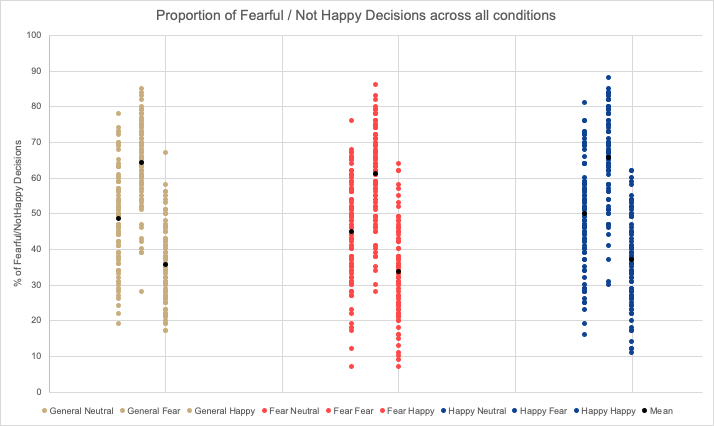


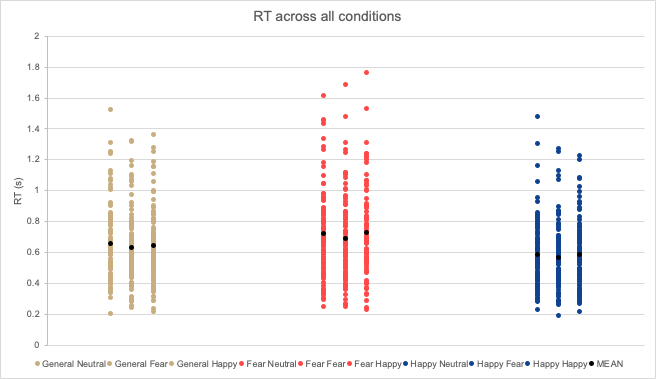


**Supplementary Figure 5**

*Q-Q plot of RT residuals.*

*
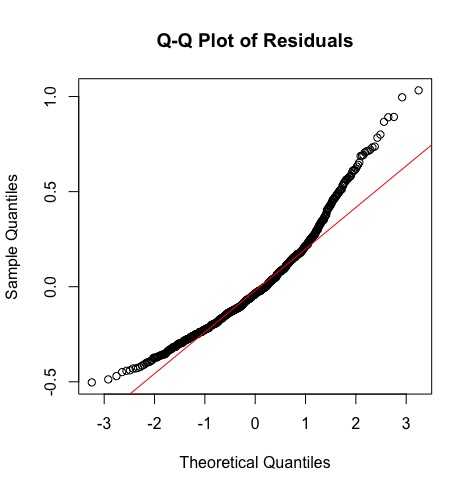
*

**Supplementary Figure 6**

*Residuals versus fitted values in RT analysis.*

*
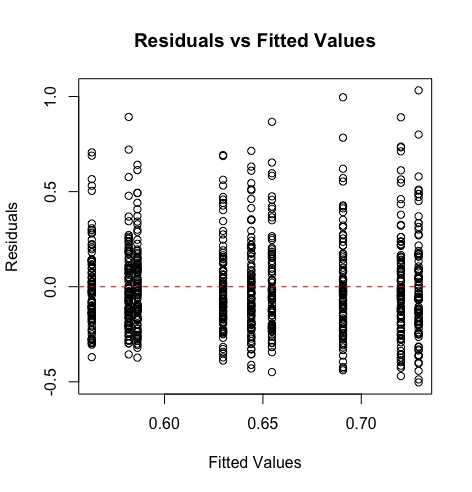
*

**Supplementary Figure 7**

*RT Residuals by condition (StimEmo and AttnEmo).*

*
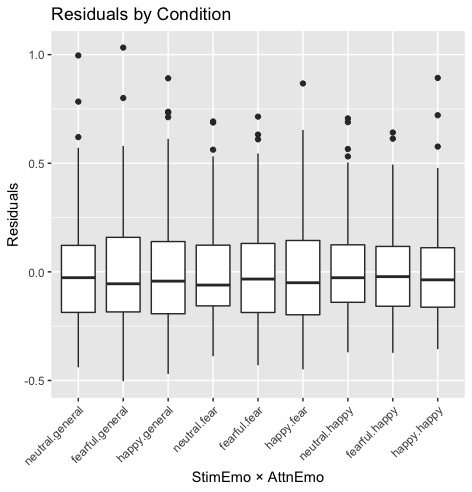
*

***Supplementary Table 2:*** *Descriptive statistics of estimated DDM parameters.*

| ***Model 1*** | ***Mean*** | ***SD*** |
| --- | --- | --- |
| *Boundary separation* | 1.412 | 0.266 |
| *Non-decision time* | 0.100 | 0.099 |
| *Starting point bias* | 0.482 | 0.047 |
| *Drift rate (Neutral StimEmo)* | 0.020 | 0.293 |
| *Drift rate (Fearful StimEmo)* | 0.546 | 0.240 |
| *Drift rate (Happy StimEmo)* | -0.401 | 0.235 |
| ***Model 4: Gen vs Fear AttnEmo*** | ***Mean*** | ***SD*** |
| *Boundary separation (Gen AttnEmo)* | 1.460 | 0.294 |
| *Boundary separation (Fear AttnEmo)* | 1.541 | 0.265 |
| *Non-decision time* | 0.078 | 0.078 |
| *Starting point bias* | 0.496 | 0.045 |
| *Drift rate (Neutral StimEmo)* | -0.083 | 0.272 |
| *Drift rate (Fearful StimEmo)* | 0.417 | 0.232 |
| *Drift rate (Happy StimEmo)* | -0.447 | 0.244 |
| ***Model 4: Gen vs Happy AttnEmo*** | ***Mean*** | ***SD*** |
| *Boundary separation (Gen AttnEmo)* | 1.489 | 0.301 |
| *Boundary separation (Happy AttnEmo)* | 1.378 | 0.234 |
| *Non-decision time* | 0.069 | 0.073 |
| *Starting point bias* | 0.478 | 0.045 |
| *Drift rate (Neutral StimEmo)* | 0.049 | 0.291 |
| *Drift rate (Fearful StimEmo)* | 0.570 | 0.251 |
| *Drift rate (Happy StimEmo)* | -0.361 | 0.242 |

*Note.* Our estimated parameters for drift rate, boundary separation, and starting point fall within the typical ranges observed in published DDM studies of perceptual decision making (Myers et al., 2022). For example, drift rates in comparable tasks generally range from approximately -1 to 1 if the prior mean is 0 and takes on a normal distribution. Boundary separation values typically lie between 0.5 to 2.0 and cannot be lower than 0, while starting point ranges from 0 to 1.0. Our parameter estimates are consistent with these ranges, indicating that the cognitive processes captured by our models align with established findings.

**Trait anxiety detailed analysis**

The covariate of anxious apprehension, as captured by PSWQ scores, was not significantly related to proportion of fearful choice, *F*(2, 186) = 2.39, *p* = .10, and RT, *F*(2, 186) = 1.61, *p* = .20. Trait anxiety as captured by DASS-21 scores was also not significantly related to proportion of fearful choice, *F*(2, 186) = 1.30, *p* = .28, and RT, *F*(2, 186) = .12, *p* = .89.

For HDDM measures, anxious apprehension was not significantly related to drift rate in Model 1, *F*(2, 186) = 0.89, *p* = .64, boundary separation from Model 4 for fear versus general-attention trials, *F*(1, 93) = 0.73, *p* = .86, as well as for happy versus general attention trials *F*(1,93) = 0.64, *p* = .93. Trait anxiety was also not significantly related to drift rate in Model 1, *F*(2, 186) = 0.89, *p* = .72, boundary separation from Model 4 for fear versus general-attention trials, *F*(1,93) = 0.28, *p* = .60, as well as for happy versus general attention trials *F*(1,93) = 0.002, *p* = .96.

**HDDM posterior predictive check (PPC) and parameter recovery result**

**Supplementary Tables 3, 4, and 5** depict the posterior predictive check (PPC) results for Model 1, Model 4 Fear- and General-attention trials, and Model 4 Happy- and General-attention trials, respectively. The table values show that there was a good match between the observed and simulated summary statistics of each model, reflecting that the winning models were able to yield data similar to the observed human data.

The parameter recovery results of Model 1, where drift rate was allowed to vary as a function of StimEmo, is depicted in **Supplementary Figure 8**. The results show that our model-fitting approach can yield decent parameter recovery. While the fitted and recovered parameters had slightly different means, there was overlap in the 95% highest density interval (HDI) range for most parameters.

The parameter recovery results of Model 4 comparing Fear- and General-attention trials, where drift rate was allowed to vary as a function of StimEmo and boundary separation was allowed to vary as a function of AttnEmo, is illustrated in **Supplementary Figure 9**. The results for the same set of analysis for Model 4 comparing Happy- and General-attention trials are depicted in **Supplementary Figure 10**. Overall, the results show that our model-fitting approach can yield good parameter recovery. Similar to Model 1, while the fitted and recovered parameters had slightly different means, there was overlap in the 95% highest density interval (HDI) range for most parameters.

**Supplementary Table 3**

*Model 1 PPC results.*

| **stat** | **observed** | **mean** | **std** | **SEM** | **MSE** | **credible** |
| --- | --- | --- | --- | --- | --- | --- |
| **accuracy** | 0.496 | 0.500 | 0.175 | 1.727e-05 | 0.031 | TRUE |
| **mean_ub** | 0.618 | 0.624 | 0.237 | 3.301e-05 | 0.056 | TRUE |
| **std_ub** | 0.504 | 0.405 | 0.173 | 0.010 | 0.040 | TRUE |
| **10q_ub** | 0.184 | 0.253 | 0.123 | 0.005 | 0.020 | TRUE |
| **30q_ub** | 0.350 | 0.367 | 0.155 | 0.000 | 0.024 | TRUE |
| **50q_ub** | 0.487 | 0.506 | 0.200 | 0.000 | 0.041 | TRUE |
| **70q_ub** | 0.676 | 0.711 | 0.275 | 0.001 | 0.077 | TRUE |
| **90q_ub** | 1.182 | 1.138 | 0.447 | 0.002 | 0.201 | TRUE |
| **mean_lb** | -0.613 | -0.603 | 0.244 | 0.000 | 0.060 | TRUE |
| **std_lb** | 0.528 | 0.402 | 0.174 | 0.016 | 0.046 | TRUE |
| **10q_lb** | 0.155 | 0.240 | 0.130 | 0.007 | 0.024 | TRUE |
| **30q_lb** | 0.332 | 0.348 | 0.163 | 0.000 | 0.027 | TRUE |
| **50q_lb** | 0.477 | 0.483 | 0.210 | 3.417e-05 | 0.044 | TRUE |
| **70q_lb** | 0.673 | 0.686 | 0.284 | 0.000 | 0.081 | TRUE |
| **90q_lb** | 1.198 | 1.111 | 0.452 | 0.008 | 0.212 | TRUE |

*Note.* “observed” depicts the value of the summary statistic of the actual data. “mean” is the mean of the summary statistics of the simulated data sets. “std” reflects how much variation there is in the summary statistic. Each of the remaining columns measure how far the summary statistic of the actual data is away from the simulated data. “SEM” stands for standard error from the mean, “MSE” stands for mean-squared error, “credible” is whether the data is in the 95% credible interval.

**Supplementary Table 4**

*Model 4 Fear- and General- attention PPC results.*

| **stat** | **observed** | **mean** | **std** | **SEM** | **MSE** | **credible** |
| --- | --- | --- | --- | --- | --- | --- |
| **accuracy** | 0.481 | 0.482 | 0.172 | 1.711e-06 | 0.029 | TRUE |
| **mean_ub** | 0.642 | 0.655 | 0.242 | 0.000 | 0.059 | TRUE |
| **std_ub** | 0.522 | 0.456 | 0.193 | 0.004 | 0.042 | TRUE |
| **10q_ub** | 0.187 | 0.240 | 0.108 | 0.003 | 0.014 | TRUE |
| **30q_ub** | 0.353 | 0.365 | 0.146 | 0.000 | 0.021 | TRUE |
| **50q_ub** | 0.502 | 0.521 | 0.200 | 0.000 | 0.040 | TRUE |
| **70q_ub** | 0.710 | 0.752 | 0.286 | 0.002 | 0.084 | TRUE |
| **90q_ub** | 1.252 | 1.233 | 0.481 | 0.000 | 0.231 | TRUE |
| **mean_lb** | -0.645 | -0.652 | 0.256 | 5.291e-05 | 0.066 | TRUE |
| **std_lb** | 0.540 | 0.455 | 0.194 | 0.007 | 0.045 | TRUE |
| **10q_lb** | 0.171 | 0.237 | 0.116 | 0.004 | 0.018 | TRUE |
| **30q_lb** | 0.343 | 0.363 | 0.159 | 0.000 | 0.026 | TRUE |
| **50q_lb** | 0.499 | 0.517 | 0.215 | 0.000 | 0.047 | TRUE |
| **70q_lb** | 0.716 | 0.749 | 0.303 | 0.001 | 0.093 | TRUE |
| **90q_lb** | 1.269 | 1.230 | 0.496 | 0.002 | 0.248 | TRUE |

**Supplementary Table 5**

*Model 4 Happy- and General- attention PPC results.*

| **stat** | **observed** | **mean** | **std** | **SEM** | **MSE** | **credible** |
| --- | --- | --- | --- | --- | --- | --- |
| **accuracy** | 0.502 | 0.506 | 0.173 | 1.803e-05 | 0.030 | TRUE |
| **mean_ub** | 0.589 | 0.604 | 0.235 | 0.000 | 0.055 | TRUE |
| **std_ub** | 0.493 | 0.419 | 0.182 | 0.005 | 0.039 | TRUE |
| **10q_ub** | 0.168 | 0.220 | 0.103 | 0.003 | 0.013 | TRUE |
| **30q_ub** | 0.323 | 0.339 | 0.142 | 0.000 | 0.020 | TRUE |
| **50q_ub** | 0.460 | 0.483 | 0.194 | 0.000 | 0.038 | TRUE |
| **70q_ub** | 0.645 | 0.695 | 0.277 | 0.002 | 0.079 | TRUE |
| **90q_ub** | 1.137 | 1.135 | 0.460 | 4.548e-06 | 0.212 | TRUE |
| **mean_lb** | -0.578 | -0.576 | 0.235 | 4.478e-06 | 0.055 | TRUE |
| **std_lb** | 0.504 | 0.412 | 0.181 | 0.008 | 0.041 | TRUE |
| **10q_lb** | 0.142 | 0.203 | 0.107 | 0.004 | 0.015 | TRUE |
| **30q_lb** | 0.306 | 0.313 | 0.145 | 5.826e-05 | 0.021 | TRUE |
| **50q_lb** | 0.449 | 0.452 | 0.197 | 1.282e-05 | 0.039 | TRUE |
| **70q_lb** | 0.640 | 0.662 | 0.277 | 0.000 | 0.077 | TRUE |
| **90q_lb** | 1.126 | 1.098 | 0.458 | 0.001 | 0.210 | TRUE |

**Supplementary Figure 8**

*Model 1 parameter recovery results.
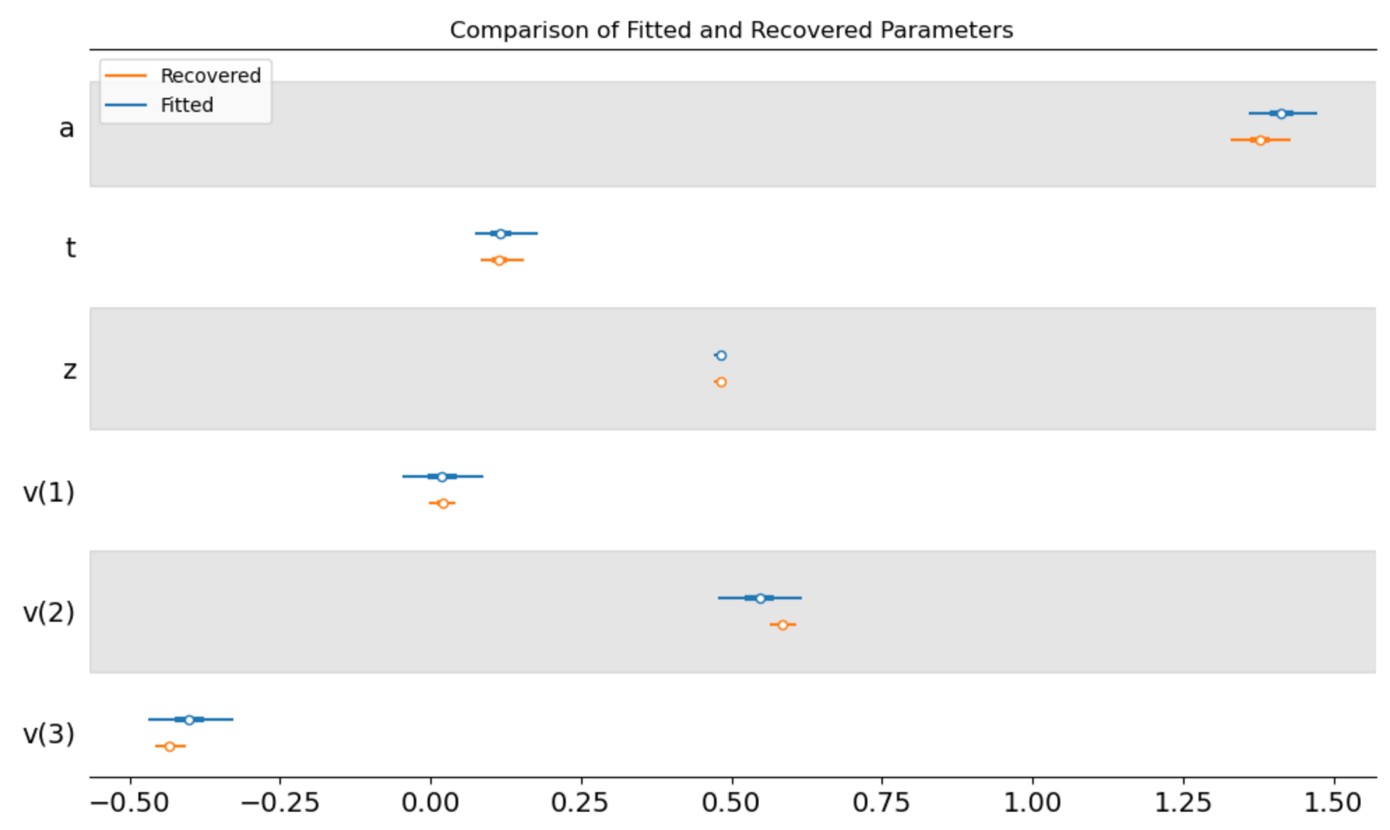
*

*Note.* The true parameter is depicted in blue and the recovered parameter is depicted in orange. White dots represent the mean with the bars indicating 95% highest density interval (HDI) range. The figure shows parameter recovery results at the group level, including six parameters, of which, the first three are subject-level parameters and last three are condition specific parameters. v(1) refers to neutral StimEmo, v(2) refers to fear StimEmo, v(3) refers to happy StimEmo,

**Supplementary Figure 9**

*Model 4 Fear- and General- attention parameter recovery results.*

*
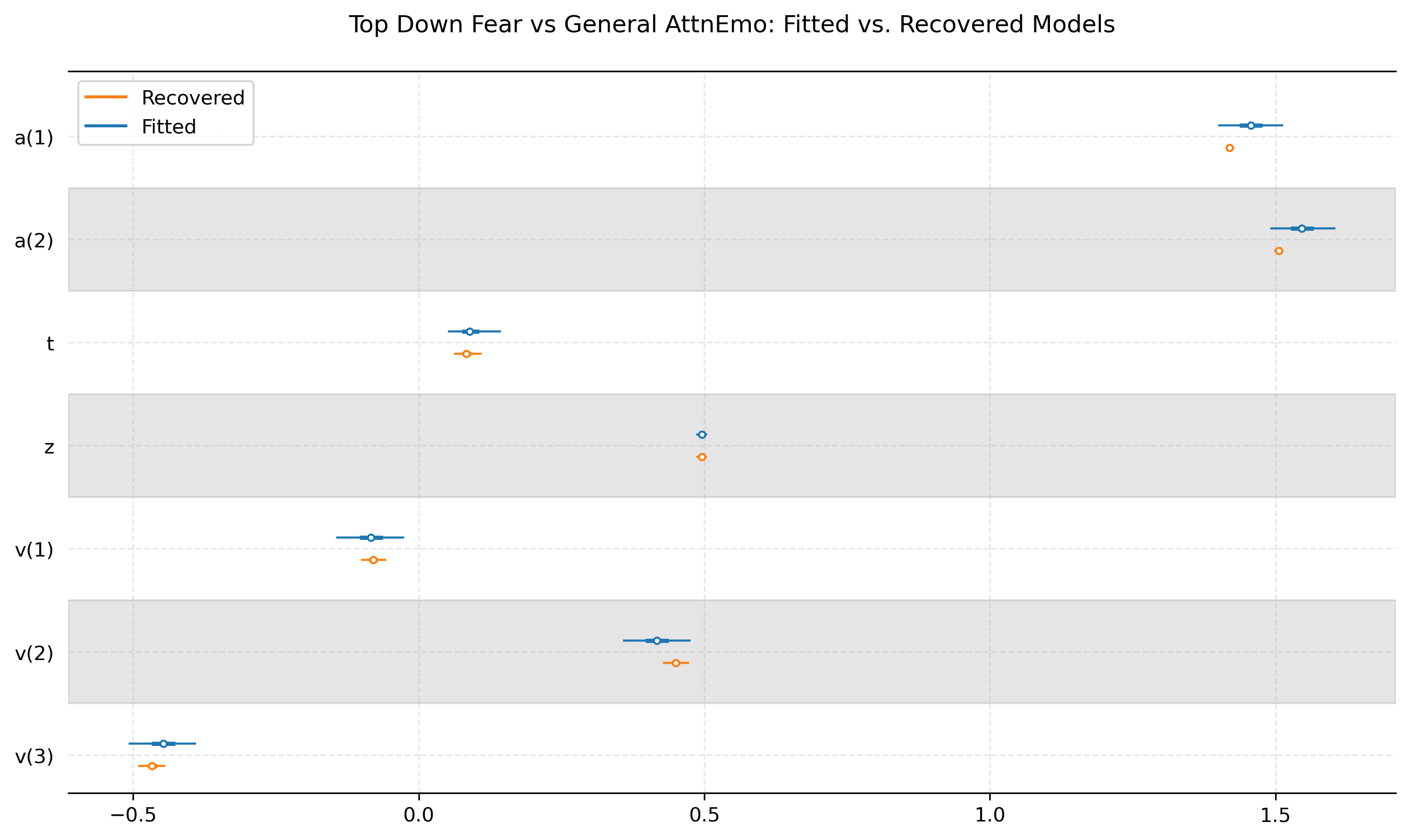
*

*Note.* The true parameter is depicted in blue and the recovered parameter is depicted in orange. White dots represent the mean with the bars indicating 95% HDI range. The figure shows parameter recovery results at the group level, including seven parameters, of which, the first two and last three are condition specific parameters. a(1) refers to General AttnEmo, a(2) refers to Fearful AttEmo. v(1) refers to neutral StimEmo, v(2) refers to fear StimEmo, v(3) refers to happy StimEmo.

**Supplementary Figure 10**

*Model 4 Happy- and General- attention parameter recovery results.*

*
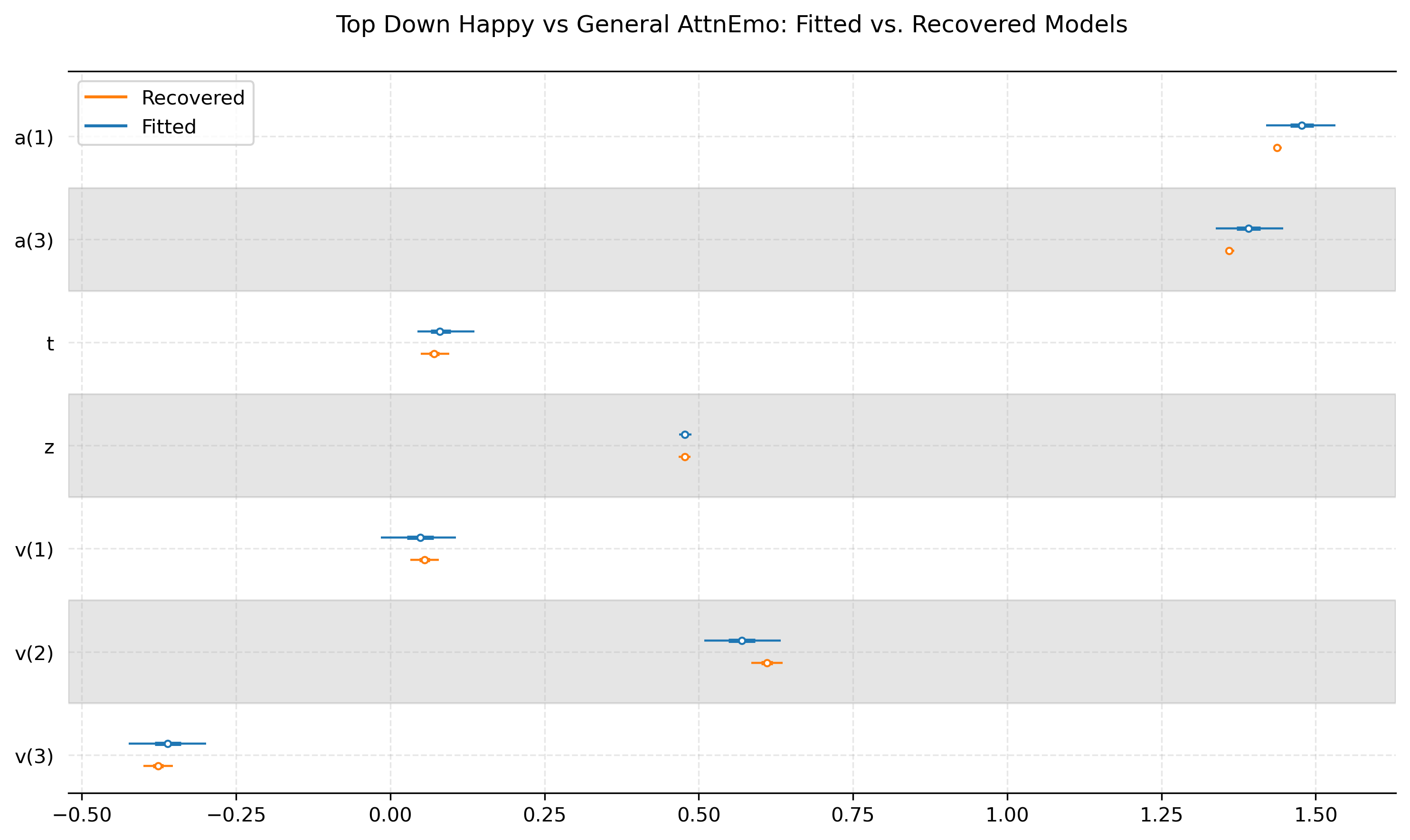
*

*Note.* The true parameter is depicted in blue and the recovered parameter is depicted in orange. White dots represent the mean with the bars indicating 95% HDI range. The figure shows parameter recovery results at the group level, including seven parameters, of which, the first two and last three are condition specific parameters. a(1) refers to General AttnEmo, a(3) refers to Happy AttEmo. v(1) refers to neutral StimEmo, v(2) refers to fear StimEmo, v(3) refers to happy StimEmo.

**Supplementary References**

Cavanagh, J. F., Wiecki, T. V., Cohen, M. X., Figueroa, C. M., Samanta, J., Sherman, S. J., & Frank, M. J. (2011). Subthalamic nucleus stimulation reverses mediofrontal influence over decision threshold. *Nature Neuroscience*, *14*(11), 1462-1467. <https://doi.org/10.1038/nn.2925>

De Kock, R., Zhou, W., Joiner, W. M., & Wiener, M. (2021). Slowing the body slows down time perception. *eLife*, *10*, e63607. <https://doi.org/10.7554/eLife.63607>

Gelman, A., Carlin, J. B., Stern, H. S., & Rubin, D. B. (2004). Bayesian Data Analysis Chapman & Hall. *CRC Texts in Statistical Science*, *136*.

Kruschke, J. K. (2018). Rejecting or accepting parameter values in Bayesian estimation. *Advances in methods and practices in psychological science*, *1*(2), 270-280.

Lawlor, V. M., Webb, C. A., Wiecki, T. V., Frank, M. J., Trivedi, M., Pizzagalli, D. A., & Dillon, D. G. (2020). Dissecting the impact of depression on decision-making. *Psychological medicine*, *50*(10), 1613-1622.

Myers, C. E., Interian, A., & Moustafa, A. A. (2022). A practical introduction to using the drift diffusion model of decision-making in cognitive psychology, neuroscience, and health sciences. *Frontiers in Psychology*, *13*, 1039172.

Pan, W., Geng, H., Zhang, L., Fengler, A., Frank, M. J., Zhang, R.-Y., & Chuan-Peng, H. (2025). dockerHDDM: A User-Friendly Environment for Bayesian Hierarchical Drift-Diffusion Modeling. *Advances in Methods and Practices in Psychological Science*, *8*(1), 25152459241298700.

Ratcliff, R. (1985). Theoretical interpretations of the speed and accuracy of positive and negative responses. *Psychological Review*, *92*(2), 212.

Ratcliff, R., Van Zandt, T., & McKoon, G. (1999). Connectionist and diffusion models of reaction time. *Psychological Review*, *106*(2), 261.

Son, J.-Y., Bhandari, A., & FeldmanHall, O. (2019). Crowdsourcing punishment: Individuals reference group preferences to inform their own punitive decisions. *Scientific Reports*, *9*(1), 1-15.

Vehtari, A., Gelman, A., Simpson, D., Carpenter, B., & Bürkner, P.-C. (2021). Rank-normalization, folding, and localization: An improved R ̂ for assessing convergence of MCMC (with discussion). *Bayesian analysis*, *16*(2), 667-718.

Wiecki, T., Sofer, I., & Frank, M. (2013). HDDM: Hierarchical Bayesian estimation of the Drift-Diffusion Model in Python [Methods]. *Frontiers in Neuroinformatics*, *7*. <https://doi.org/10.3389/fninf.2013.00014>
